# Study of the Binding Energies between Unnatural Amino Acids and Engineered Orthogonal Tyrosyl-tRNA Synthetases

**DOI:** 10.1038/srep12632

**Published:** 2015-07-29

**Authors:** Wei Ren, Tan M. Truong, Hui-wang Ai

**Affiliations:** 1 Department of Chemistry, University of California-Riverside, 501 Big Springs Road, Riverside, California 92521, United States; 2 Cell, Molecular, and Developmental Biology Graduate Program, University of California-Riverside, Riverside, California 92521, United States

## Abstract

We utilized several computational approaches to evaluate the binding energies of tyrosine (Tyr) and several unnatural Tyr analogs, to several orthogonal aaRSes derived from *Methanocaldococcus jannaschii* and *Escherichia coli* tyrosyl-tRNA synthetases. The present study reveals the following: (1) AutoDock Vina and ROSETTA were able to distinguish binding energy differences for individual pairs of favorable and unfavorable aaRS-amino acid complexes, but were unable to cluster together all experimentally verified favorable complexes from unfavorable aaRS-Tyr complexes; (2) MD-MM/PBSA provided the best prediction accuracy in terms of clustering favorable and unfavorable enzyme-substrate complexes, but also required the highest computational cost; and (3) MM/PBSA based on single energy-minimized structures has a significantly lower computational cost compared to MD-MM/PBSA, but still produced sufficiently accurate predictions to cluster aaRS-amino acid interactions. Although amino acid-aaRS binding is just the first step in a complex series of processes to acylate a tRNA with its corresponding amino acid, the difference in binding energy, as shown by MD-MM/PBSA, is important for a mutant orthogonal aaRS to distinguish between a favorable unnatural amino acid (unAA) substrate from unfavorable natural amino acid substrates. Our computational study should assist further designing and engineering of orthogonal aaRSes for the genetic encoding of novel unAAs.

In most organisms, 61 trinucleotide codons encode for the 20 canonical amino acids^1^. An additional three codons (UAA, UAG, and UGA) are nonsense “stop” codons that trigger the termination of ribosomal protein synthesis^2^. Proteins can undergo posttranslational modifications to further derivatize the canonical 20 amino acids, thereby increasing complexity and biological control to achieve sophisticated cellular functions^3^. In the 1960s, nonsense suppression bacterial strains were discovered to express additional tRNAs that recognize the stop codons UAG (amber suppressor), UAA (ochre suppressor), or UGA (opal suppressor)^1^,^4^. There are also exceptional examples in which uncommon amino acids, such as pyrrolysine or selenocysteine, can be prompted for ribosomal peptide synthesis in response to UAG or UGA codons, respectively^5^,^6^,^7^. Furthermore, in the past few decades, research has expanded the possibilities of protein structures and functions through an expansion of the genetic codes^8^,^9^. Additional pairs of aminoacyl-tRNA synthetases (aaRSes) and the corresponding tRNAs that do not cross-react with endogenous tRNAs, aaRSes, and amino acids, have been engineered and expressed in living bacteria, yeast, mammalian cells and several model multicellular organisms^8^,^9^,^10^,^11^. These aaRSes and tRNAs have been pre-engineered to use unnatural amino acids (unAAs) as their substrates, thereby affording the genetic encoding of unAAs in living cells and organisms. This genetic code expansion technology has since produced modified proteins with site-specific incorporation of a large array of unAAs^8^, such as fluorescent amino acids^12^,^13^, biophysical probes^14^,^15^,^16^, photocrosslinkers^17^,^18^,^19^, reactive chemical handles for bioorthogonal reactions^20^,^21^,^22^, photocaged amino acids^23^,^24^,^25^,^26^,^27^,^28^, and amino acids identical to or mimicking posttranslational modifications^29^,^30^,^31^,^32^. This method has now been broadly utilized not only to create proteins with enhanced and novel properties, but also to develop novel therapeutics and investigate protein structure and function^8^.

Engineered orthogonal tRNAs are typically adapted into an organism of interest from evolutionarily distant species to lower the likelihood of cross-recognition by endogenous aaRSes^8^. Even so, the corresponding aaRSes, with some exceptions involving pyrrolysyl-tRNA synthetases^33^, would still have to undergo extensive protein engineering to switch their substrate preference from a native amino acid to an unAA. This bioengineering procedure, which typically involves several rounds of positive and negative selection, is laborious and time-consuming, and requires considerable expertise^8^. The number of residues that can be simultaneously mutated is often limited to 5 or 6, due to technical limitations with the molecular biology^34^. Hence, it is often not trivial to derive orthogonal aaRSes for unAA substrates that are very different from the enzymes’ native substrates. New strategies are currently being explored to circumvent this challenging positive and negative selection process. For example, some existing orthogonal aaRSes can use several different unAAs as their substrates, so their polyspecificity has been exploited for the genetic encoding of new unAAs^34^,^35^,^36^. This substrate promiscuity is not problematic, however, because the experimenter controls the unAA(s) supplemented into the culture medium for any given experiment.

It may also be prudent to computationally design aaRSes for unAAs because computational methods have been routinely utilized to study enzyme-substrate interactions^37^. Previously, Wang *et al.* and Datta *et al.* computationally estimated the binding energies of natural *E. coli* phenylalanyl- and methionyl-tRNA synthetases to several unnatural phenylalanine or methionine analogues, respectively, and compared these values to the experimental activities of these enzyme-substrate pairs^38^,^39^. Using this method, they were able to genetically incorporate several unAAs into proteins using auxotrophic *E. coli*, but could not achieve site-specificity due to cross-reactivity issues. Computational studies on engineered orthogonal aaRSes and unAAs are scarce. In a previous work, Zhang *et al.* reported a clash opportunity progressive (COP) method to identify a possible mutant of *M. jannaschii* tyrosyl-tRNA synthetase (*Mj*TyrRS) for preferential binding to *O*-methyl-L-tyrosine over Tyr^40^. In another work, Sun *et al.* docked *p*-acetyl-L-phenylalanine (AcF) into 60 different *Mj*TyrRS mutants to identify possible mutations benefiting enzyme-substrate binding^41^. The full capability of the methods by Zhang *et al.* and Sun *et al.* in designing orthogonal aaRSes for unAAs is still unclear. Each study focused on only one unAA, and the mutant aaRSes derived from their computational studies were not experimentally tested; although their computationally derived sequences showed some similarities to orthogonal aaRSes previously derived from experimental studies by Schultz *et al.*^10^,^42^.

Aminoacylation of tRNAs by aaRSes is a complex, multi-step process^43^. In general, aaRS is bound by a specific amino acid substrate, which is subsequently activated through an adenylation reaction with ATP. The activated amino acid is then transferred to the 3′ end of an aaRS-bound tRNA by releasing the attached AMP molecule, consequently producing a charged tRNA. A computational model depicting this entire process is very difficult to produce. Like many other enzyme-substrate studies^37^, we presume that the ability for an amino acid to bind a particular aaRS is very important for establishing their enzyme-substrate relationship. To achieve the goal of computationally designing orthogonal aaRSes for unAAs, it might not be necessary to accurately estimate the absolute binding energy and binding affinity of an aaRS/unAA pair; however, it is critical to identify computational parameters that can group favorable and unfavorable aaRS-amino acid complexes. Herein, we report our evaluation of several computational methods for scoring binding energies of a number of aaRS-amino acids complexes. These benchmark experiments were performed with complexes of orthogonal *Mj*TyrRS or *Ec*TyrRS mutants bound to their experimentally verified unAA substrates, and compared to Tyr—the natural substrate for wild-type *Mj*TyrRS and *Ec*TyrRS. We compared the results of several popular computational methods, including AutoDock Vina^44^, ROSETTA^45^, and Molecular Mechanics/Poisson–Boltzmann Surface Area (MM/PBSA)^46^. We performed MM/PBSA binding energy scoring based on a 10-ns Molecular Dynamics (MD) simulation, and direct MM-PBSA scoring based on single energy-minimized structures. These tested methods, which required varying amounts of computational resources, yielded different capabilities for grouping favorable and unfavorable aaRS-amino acid interactions. Moreover, we analyzed the factors contributing to amino acid recognition. In particular, a polyspecific *Ec*TyrRS mutant was studied for its capacity to utilize several different unAAs as its enzymatic substrates^36^,^47^.

## Methods

### Preparation of aaRS-amino acid complexes

We computationally studied seven aaRS-amino acid complexes. The following two X-ray crystal structures were downloaded from Protein Data Bank (PDB): *Mj*TyrRS-derived *p*-acetylphenylalanyl-tRNA synthetase (*Mj*AcFRS) bound with AcF (PDB 1ZH6)^48^, and *Mj*TyrRS-derived 3*-*iodotyrosyl-tRNA synthetase (*Mj*IoYRS) bound with 3*-*iodo-L-tyrosine (IoY) (PDB 2ZP1)^49^. These two complexes were cleaned by removing water molecules, co-crystallized ions, and non-amino acid ligands. Hydrogen atoms of the amino acid ligands were added in VEGA ZZ^50^. To derive the complex structures of proteins bound with Tyr, the side chains of the unAA ligands in the above two complexes were manually edited in VEGA ZZ and combined with the corresponding protein coordinates by matching the coordinates of the unchanged ligand atoms. This process generated two additional aaRS-amino acid complexes: *Mj*AcFRS bound with Tyr, and *Mj*IoYRS bound with Tyr. No X-ray crystal structure is available for the polyspecific synthetase (*Ec*PolyRS) derived from *Ec*TyrRS. Based on the X-ray crystal structure of the wild-type *Ec*TyrRS (PDB 1X8X)^51^, we used SWISS-MODEL^52^ to perform homologous modeling of the *Ec*PolyRS structure. The coordinates of Tyr in 1X8X were combined with the modeled protein coordinates to derive an *Ec*PolyRS-Tyr complex. We also manually edited Tyr in VEGA ZZ to derive coordinates for the unAAs, *p*-iodophenylalanine (IoF) and AcF. They were combined with the modeled *Ec*PolyRS coordinates to derive two additional complexes: *Ec*PolyRS bound with IoY and *Ec*PolyRS bound with AcF.

Further relaxation of these complexes was achieved with GROMACS-4.6.5^53^,^54^. The force field for proteins was set to AMBER99SB^55^. ACPYPE^56^ was used to treat ligands based on Generalized Amber Force Field (GAFF)^57^,^58^. The complexes were immersed in a dodecahedron box of SPC/E water molecules. The water box was extended 1 nm from solute atoms in all directions. Counter ions, such as Na^+^ and Cl^–^, were added to neutralize the systems. Particle mesh Ewald (PME) was used to treat the long-range electrostatic interactions in molecular mechanics (MM) energy minimization. The systems were minimized by using the steepest descent algorithm. The minimization was stopped either at 50 000 steps or until the maximum force was smaller than 10.0 kJ/mol.

### Binding energy scoring with Autodock Vina and ROSETTA

The energy score function embedded in Autodock Vina 1.1.2^44^ was used to assess the binding free energies of all complexes. The pdbqt files of proteins and ligands were prepared in AutoDockTools^59^ from the above-mentioned complexes. Polar hydrogens were added and the binding free energies were calculated using the embedded “score only” option in Autodock Vina.

Coordinates of proteins and ligands were separated in PyMol. We followed a previously reported procedure to score aaRS-amino acid complexes using ROSETTA 3.5^45^. The interface energy term was used in this study to evaluate the binding.

### Molecular Dynamics simulations

Molecular Dynamics (MD) simulations were performed in Gromacs-4.6.5^53^,^54^. The solvated and MM-energy-minimized ligand-protein complexes were heated to 300 K during a 100 ps constant volume simulation with 2 fs time step. The pressure was then equilibrated to 1 atm during a 100 ps isothermal-isobaric NPT simulation with 2 fs time step. All heavy atoms were position-restrained with a force constant of 1000 kJ•mol^−1^•nm^−2^. Simulations were performed for 10 ns with a time step of 2 fs. The temperature and pressure were maintained at 300 K and 1 atm using the V-rescale temperature and Parrinello-Rahman pressure coupling method, respectively. The time constants for the temperature and pressure coupling were set at 0.1 ps and 2 ps, respectively. Short-range, non-bonded interactions were computed for the atom pairs within the 9 Å cutoff. Long-range electrostatic interactions were calculated using a PME summation method with fourth-order cubic interpolation and 1.6 Å grid spacing. All bonds were constrained using the parallel LINCS method. Xmgrace was used to plot the data and graphs generated from Gromacs.

### MM/PBSA binding energy calculation

We used g_mmpbsa to estimate MM/PBSA binding energies^60^. The average binding energy was calculated from 100 snapshots extracted every 50 ps from the MD trajectories between 5 and 10 ns. The non-polar solvation energy was calculated based on the SASA model. The vacuum and solvent dielectric constants were set at 1 and 80, respectively. The solute dielectric constant was set at 2. The entropy term was not included in our binding energy calculation. A bootstrap analysis was performed to obtain standard errors. To calculate the binding energy based on single snapshots, we followed all of the abovementioned procedure, except that MM-energy-minimized aaRS-unAA complexes were directly utilized for energy calculations without any MD treatment.

## Results and Discussion

### Selection and preparation of aaRS-amino acid complexes

**Figure 1:**
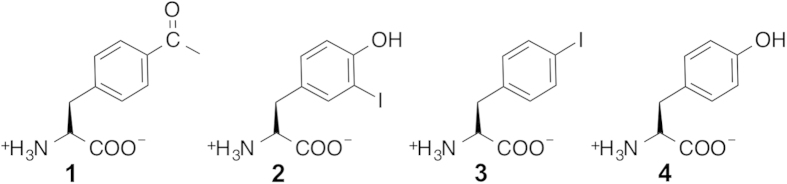
Chemical structures of natural and unnatural amino acids used in this study. (**1**: *p*-acetyl-L-phenylalanine, AcF; **2**: 3-iodo-L-tyrosine, IoY; **3**: *p*-iodo-L-phenylalanine, IoF; and **4**: L-tyrosine, Tyr).

A very large number of orthogonal aaRSes have been derived from *Mj*TyrRS and *Ec*TyrRS^8^, which are currently widely utilized for the genetic encoding of unAAs in bacterial and eukaryotic cells, respectively. We examined available co-crystal structures of *Mj*TyrRS mutants with unAAs and decided to use two complexes in our study: *Mj*AcFRS bound with AcF, and *Mj*IoYRS bound with IoY. AcF has a side-chain carbonyl group for H-bond formation with the corresponding aaRS, whereas the IoY side chain can interact with the aaRS through both H-bonding and non-H-bonding van der Waals interactions (Fig. 1). For our study, we also selected an *Ec*TyrRS-derived polyspecific synthetase, *Ec*PolyRS, which was originally engineered for the genetic encoding of IoF^11^. In addition to IoF, we later found that *Ec*PolyRS was also capable of using several other unAAs, including AcF, as its substrate^36^,^47^. Because no X-ray crystal structure of *Ec*PolyRS is available, we used the wild-type *Ec*TyrRS 3D-structure as the template for homologous modeling of *Ec*PolyRS. The manually edited coordinates of unAAs, IoF and AcF (Fig. 1), were next combined with the modeled protein structure. The side chain of IoF is expected to interact with *Ec*PolyRS mainly through non-H-bonding van der Waals interactions, while the carbonyl group of AcF would act as an excellent H-bond donor. We also modeled Tyr into these aforementioned aaRS structures in order to computationally evaluate and compare the binding energies of these aaRSes to Tyr. All three selected aaRSes have an excellent capacity for discriminating unAAs from Tyr, as shown from previous protein-expression experiments by the lack of Tyr usage as an enzymatic substrate at physiological concentrations^11^,^42^,^49^. Because our ultimate goal is to computationally design orthogonal aaRSes for unAAs, and currently, it is difficult to model water molecules at the protein/ligand interfaces to effectively mediate interactions, we removed water molecules from these complex structures. All abovementioned amino acid-aaRS complexes were subjected to relaxation through a standard MM energy minimization process.

### Binding energy scoring with AutoDock Vina and ROSETTA

In order to achieve the computational design of orthogonal aaRSes for unAAs, it is crucial to predict their interaction modes and ultimately differentiate the interactions of aaRSes between different amino acid ligands. In this present study, we do not evaluate strategies for protein randomization and binding pose searching. Instead, we mainly focus on approaches to evaluate binding affinities of aaRSes and amino acids. Energy scoring functions implemented in docking programs are usually designed to minimize computing costs, and thus, they can be utilized to evaluate large numbers of protein–ligand complexes^44^,^61^. We first utilized AutoDock Vina, a popular molecular docking suite that includes an Amber-force-field-based scoring function, to evaluate the interactions of our selected aaRSes with corresponding unAAs and Tyr. The estimated free energies of binding were all within the range of −5.68 to −7.09 kJ/mol (Table 1). Although the estimated binding free energies between aaRSes and unAAs were typically lower than that between aaRSes and Tyr, the differences were minimal. The binding free energies scored with AutoDock Vina, for both *Mj*IoYRS-IoY and *Ec*PolyRS-IoF, were only different from their corresponding aaRS-tyrosine complexes by 5%. Larger differences (~15%) were observed for *Mj*AcFRS-AcY and *Ec*PolyRS-AcF. The binding free energies for the examined four favorable aaRS-unAA complexes were −6.61 ± 0.49 kJ/mol, whereas the binding energies for the three unfavorable aaRS-Tyr complexes were −6.09 ± 0.39 kJ/mol. This method failed to confidentially distinguish favorable interaction from unfavorable interactions. It is worthwhile to note that the numbers in Table 1 were derived by scoring single poses from X-ray crystal structures or homologous models, and the gaps were not improved by performing protein-ligand docking with flexible aaRS side chains.

We next turned to ROSETTA, another popular suite of programs widely used for protein structure prediction, protein design, and protein-protein and protein-ligand docking^62^. We scored the interface energies of various aaRSes-amino acid complexes. The estimated interface energies in ROSETTA energy units (REU) are shown in Table 1. This method was generally capable of identifying the binding energy differences of aaRSes to their real unAA substrates and Tyr; and these differences were within the range of 19% to 44%. However, when all estimated interface energies were analyzed together, there was no obvious threshold to differentiate between favorable and unfavorable interactions, as defined by wet lab results. For example, the interface energy score for the unfavorable *Mj*IoYRS-Tyr complex (−11.21 REU) was even lower than that for the favorable *Mj*AcFRS-AcF complex (−10.72 REU). Our data indicates that ROSETTA might not be very reliable to predict whether an amino acid is a true substrate of a particular aaRS.

### Binding energy estimation by MD-MM/PBSA or direct MM/PBSA

**Figure 2:**
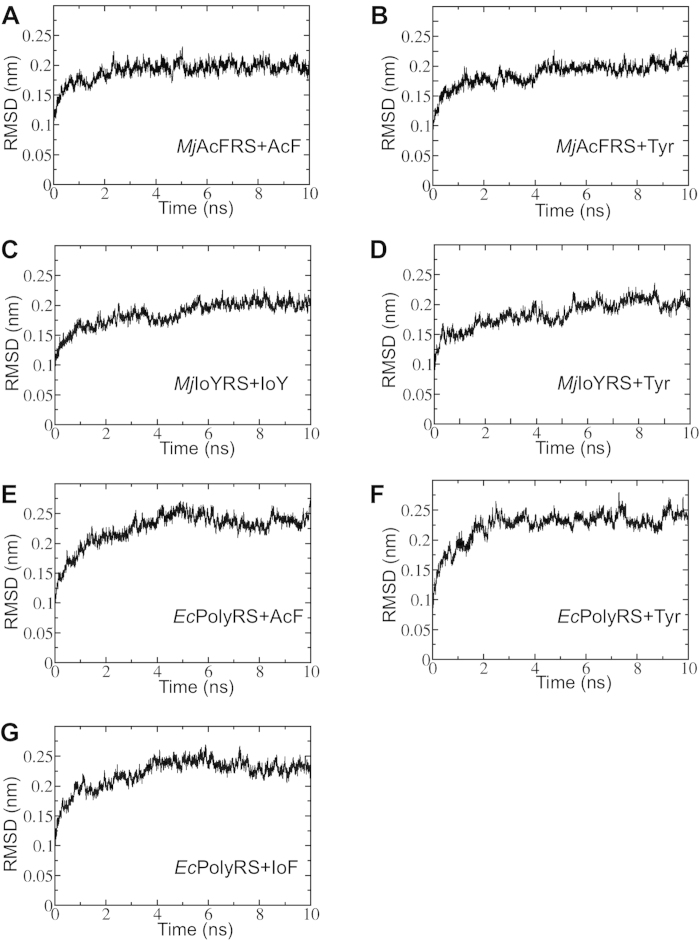
The RMSD values in the MD trajectories of the seven studied aaRS-amino acid complexes.

Compared to energy scoring functions implemented in docking programs, free-energy simulation techniques, such as MD-MM/PBSA, are known to have better accuracy for binding energy ranking^61^. However, this gain is accompanied by a much higher computational cost. We performed 10-ns MD simulations for each protein-ligand complex. We monitored the root-mean-square deviation (RMSD) values of the whole complexes and observed that they typically reached a plateau after the first 3–4 ns (Fig. 2). We next selected 100 equal-interval snapshots between 5 ns and 10 ns of each simulation to estimate binding free energies for each aaRS-amino acid complex. Considering that the aaRS-amino acid interfaces are moderately charged, we used a dielectric constant of 2 to estimate the energy values^63^. Previous studies also showed that the conformational entropy was only important for predicting absolute binding free energies but not important for ranking the binding affinities of similar substrates^64^. Hence, in order to minimize computational costs, we did not include the entropy term in our calculations. The estimated binding energies for the aaRSes and their favorable unAA substrates were within the range of −15.35 to −19.16 kcal/mol, whereas the estimated binding energies for the aaRSes and their unfavorable Tyr substrate were within the range of −9.82 to −10.56 kcal/mol (Table 2), illustrating a distinct gap between these two groups of values. The average binding energy for the former group was −16.61 ± 1.76 kcal/mol, whereas the latter group was −10.19 ± 0.37 kcal/mol. Subjecting these two groups to a two-tailed test yields a p-value of 0.004, indicating a significant difference.

It is well accepted that MD simulation improves energy calculations by using conformational sampling, but comes at the cost of significant computational resources, thereby making MD-MM/PBSA evaluations of a large number of aaRS-amino acid complexes infeasible^61^. We next utilized MM/PBSA to directly score single energy-minimized structures of the seven aaRS-amino acid complexes^65^. The results (Table 2), derived from a much-reduced computing cost, were slightly different from energy values from sampling MD trajectories, but still useful in grouping favorable aaRS-aaRS complexes from unfavorable ones. The estimated binding energies for these favorable complexes were within the range of −16.48 to −21.87 kcal/mol, whereas the numbers for these unfavorable ones were within the range of −9.33 to −11.13 kcal/mol. The average value for the former group was −18.83 ± 2.36 kcal/mol, whereas the latter group was −10.14 ± 0.91 kcal/mol. A two-tailed test still showed a significant difference (p = 0.002) between the two groups.

Scoring functions of docking softwares use various approximations to increase computational efficiency^61^. These methods are designed for screening a large number of mutants with reasonable speeds, but at the cost of accuracy. MM/PBSA uses a more-rigorous scoring function, generally leading to better prediction accuracy^65^. Considering this and based on our results, we suggest using direct MM/PBSA scoring to re-evaluate top hits of orthogonal aaRS designs from docking programs, such as AutoDock Vina and ROSETTA. Moreover, for the few top-ranked candidates in single-structure MM/PBSA scoring experiments, it may be desirable to perform MD and MM/PBSA rescoring based on snapshots of MD trajectories to increase the accuracy. This combinatorial approach, which balances computational costs and prediction accuracy, has the potential to accelerate the engineering of orthogonal aaRS for the genetic encoding of unAAs.

### Binding modes of aaRS-unAA complexes

**Figure 3:**
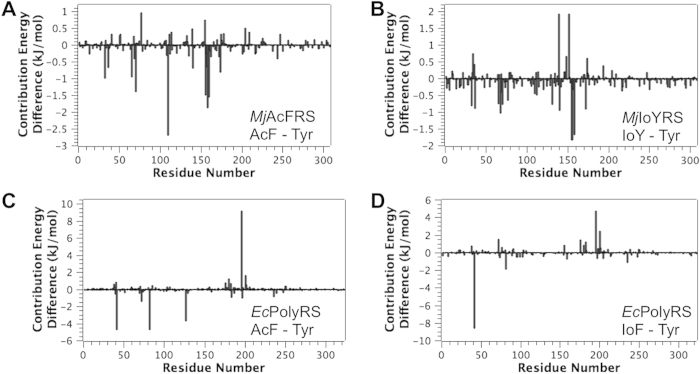
The contributions of individual amino acid residues of aaRSes to the total binding energies, shown as the energy contribution differences between the indicated aaRS-unAA complexes and aaRS-Tyr complexes. Negative values indicate a stabilization effect for aaRS-unAA interactions or a destabilization effect for aaRS-Tyr interactions, whereas positive values indicate a destabilization effect for aaRS-unAA interactions or a stabilization effect for aaRS-Tyr interactions.

**Figure 4:**
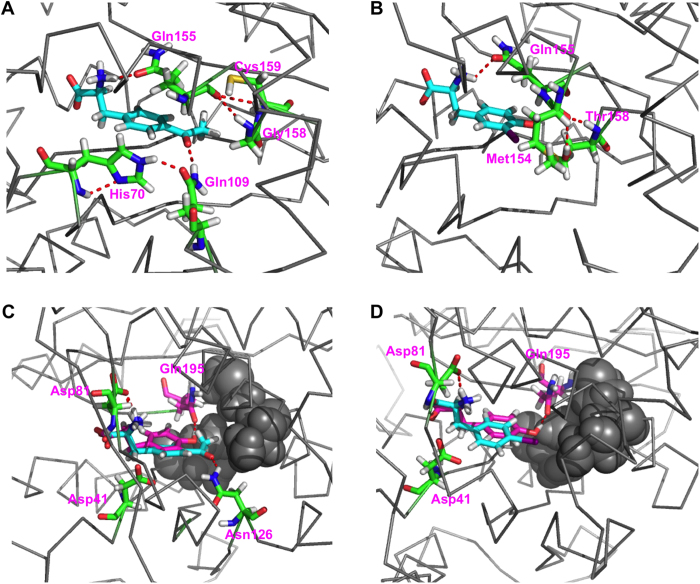
MD-averaged structures showing the active sites of the studied aaRSes and unAA complexes. (**A**): *Mj*AcFRS bound with AcF; (**B**): *Mj*IoYRS bound with IoY; (**C**): *Ec*PolyRS bound with AcF; and (**D**): *Ec*PolyRS bound with IoF). Ligands are shown as cyan sticks. Residues important for substrate specificity are shown as green sticks. In panels C and D, Tyr ligands are shown as magenta sticks for comparison. Ile37, Ser182, and Met183 of *Ec*PolyRS are shown as gray balls.

The first step of tRNA aminoacylation involves the interaction of an amino acid substrate to the aaRS, which is often the initial focus of engineering orthogonal aaRSes because its potential interaction with the natural Tyr substrate has to be minimized. Compared to co-crystal structures or structures derived from molecular modeling, MD-MM/PBSA studies can provide information on the dynamics and energy contributions for aaRS-amino acid recognition. We analyzed the contributions of individual amino acid residues of aaRSes to the total binding energies of all studied aaRS-amino acid complexes (Fig. 3). We also averaged MD structures from the MD trajectories to derive aaRS-amino acid complex structures (Fig. 4). We identified His70, Gln109, Gln155, Gly158, and Cys159 to be important for maintaining the interaction of *Mj*AcFRS to AcF versus Tyr (Figs 3A and 4A). Gln109 forms a H-bond to the carbonyl group of AcF, but not to Tyr. Gly158 and Cys159 form non-H-bond van der Waals packing interactions with the methyl group of AcF. His70 and Gln155 interact with residues 109, 158, and 159 to further stabilize the *Mj*AcFRS-AcF complex. Similarly, we found that Met154, Gln155, and Thr158 are critical for establishing packing of *Mj*IoYRS to IoY versus Tyr (Figs 3B and 4B).

Three residues in the amino acid-binding pocket of *Ec*PolyRS are different from the corresponding residues of wild-type *Ec*TyrRS: Ile37, Ser182, and Met183. Surprisingly, no strong interaction is conferred by these mutations to differentiate AcF and IoF from Tyr. Our MD study suggests that the relative interacting position of Tyr in *Ec*PolyRS is slightly different from that of AcF or IoF. The hydroxyl group of Tyr is located toward Gln195 to form a H-bond, which contributes to the stabilization of the *Ec*PolyRS-Tyr complex (Figs 3C,D and 4C,D). However, this twist significantly destabilizes the interaction with Asp81 and Asp41, consequently yielding an energy-disfavored complex as a whole. In comparison, AcF and IoF occupy the binding pocket in a way similar to Tyr in wild-type *Ec*TyrRS. They have more favorable interactions to Asp81 and Asp41. This phenomenon might explain the polyspecificity of *Ec*PolyRS, which has been shown to use at least 14 different unAAs as its enzymatic substrate^36^,^47^. These unAAs likely interact with *Ec*PolyRS in a direction similar to that of AcF and IoF, but not to Tyr; whereas no strong side-chain recognition is required to stabilize these *Ec*PolyRS-unAA complexes. We also observed a H-bond between Asn126 and the carbonyl group of AcF, but such stabilization does not exist in the *Ec*PolyRS-IoF complex, and likely, it does not exist in many other *Ec*PolyRS-unAA complexes considering the structural diversity of these 14 different unAAs^36^,^47^.

## Summary

We performed computational studies to evaluate the binding energies of several aaRS-amino acid complexes. Using orthogonal aaRS-unAA pairs whose strong interactions have been previously reported in experimental studies, we compared the accuracy of AutoDock Vina, ROSETTA, MM/PBSA, and MD-MM/PBSA in terms of grouping favorable and unfavorable interactions based on estimated binding free energies. We found that the most accurate grouping was derived from MM/PBSA based on either 10-ns MD trajectories or single energy-minimized structures. As such, we suggest using MM/PBSA to re-score top-hit poses produced by other faster, but less-accurate programs, in future aaRS-designing experiments. We also compared the binding models of the studied aaRSes to unnatural and natural amino acids. In general, the aaRSes established new H-bonds, or non-H-bond van der Waals interactions, to stabilize their unAA substrates. Moreover, they may adopt conformations to largely destabilize their interactions to the native Tyr substrate, as shown in the twisted interactions between *Ec*PolyRS and Tyr. We hope to use these results to guide future designing and development of new aaRSes, and to extend the capability of the genetic code expansion technology to many new unAAs.

## Additional Information

**How to cite this article**: Ren, W. *et al.* Study of the Binding Energies between Unnatural Amino Acids and Engineered Orthogonal Tyrosyl-tRNA Synthetases. *Sci. Rep.* **5**, 12632; doi: 10.1038/srep12632 (2015).

## Figures and Tables

**Table 1:** Estimated binding free energies using AutoDock Vena and ROSETTA for the seven tested aaRS-amino acid complexes.

Proteins		*Mj*AcFRS		*Mj*IoYRS		*Ec*PolyRS		
Amino acids		AcF	Tyr	IoY	Tyr	IoF	AcF	Tyr
**Energy Scores**	AutoDock Vina (kcal/mol)	−7.09 [1.15]^a^	−6.15	−6.85 [1.05]^a^	−6.54	−5.95 [1.05]^a^	−6.56 [1.15]^a^	−5.68
	ROSETTA (REU)	−10.72 [1.20]^a^	−8.94	−16.12 [1.44]^a^	−11.21	−13.22 [1.19]^a^	−13.27 [1.20]^a^	−11.08

^a^Ratios of the estimated binding free energies for the indicated aaRS-unAA complexes to the binding free energies of the corresponding aaRS-Tyr complexes.

**Table 2:** Calculated binding energies using MD-MM/PBSA or direct MM/PBSA for the seven aaRS-amino acid complexes.

		∆*E*_vdw_ a	∆*E*_ele_ a	∆*G*_ps_^a^	∆*G*_SASA_^a^	∆*G*_total_^a^,^b^
*Mj*AcFRS + AcF	MD-MM/PBSA	−30.56 ± 0.27	−26.12 ± 0.30	44.45 ± 0.22	−3.11 ± 0.01	−15.35 ± 0.22
	direct MM/PBSA	−30.08	−27.73	43.49	−3.24	−17.56
*Mj*AcFRS + Tyr	MD-MM/PBSA	−25.61 ± 0.25	−23.46 ± 0.30	41.68 ± 0.21	−2.79 ± 0.01	−10.17 ± 0.23
	direct MM/PBSA	−23.72	−25.99	43.31	−2.92	−9.33
*Mj*IoYRS + IoY	MD-MM/PBSA	−30.08 ± 0.29	−26.49 ± 0.33	43.09 ± 0.31	−2.97 ± 0.01	−16.45 ± 0.26
	direct MM/PBSA	−30.15	−31.61	42.71	−2.81	−21.87
*Mj*IoYRS + Tyr	MD-MM/PBSA	−24.77 ± 0.25	−25.48 ± 0.31	43.18 ± 0.24	−2.73 ± 0.01	−9.82 ± 0.25
	direct MM/PBSA	−23.54	−31.54	46.61	−2.66	−11.13
*Ec*PolyRS + IoF	MD-MM/PBSA	−26.98 ± 0.28	−30.01 ± 0.31	40.76 ± 0.38	−2.93 ± 0.01	−19.16 ± 0.32
	direct MM/PBSA	−30.19	−43.17	56.82	−2.88	−19.41
*Ec*PolyRS + AcF	MD-MM/PBSA	−30.07 ± 0.24	−31.87 ± 0.28	49.55 ± 0.32	−3.10 ± 0.01	−15.49 ± 0.25
	direct MM/PBSA	−29.76	−47.76	64.09	−3.05	−16.48
*Ec*PolyRS + Tyr	MD-MM/PBSA	−23.64 ± 0.32	−31.41 ± 0.35	47.17 ± 0.28	−2.68 ± 0.01	−10.56 ± 0.24
	direct MM/PBSA	−25.99	−41.69	60.54	−2.61	−9.75

^a^All values are given in kcal/mol, and MD-MM/PBSA values are given as average  ±  S.D.

^b^The total of van der Waals interaction energy (*∆E*_vdw_), electron static energy (*∆E*_ele_), and polar (∆*G*_ps_) and nonpolar (*∆G*_SASA_) solvation energy.
